# A case of community‐acquired pneumonia caused by *Bacillus subtilis* subsp. *natto* in an immunocompetent patient

**DOI:** 10.1002/rcr2.1384

**Published:** 2024-05-14

**Authors:** Tetsuo Tani, Tomohiro Takehara, Kota Ishioka, Ayumi Yoshifuji, Kotaro Aoki, Saeko Takahashi

**Affiliations:** ^1^ Department of Pulmonary Medicine Tokyo Saiseikai Central Hospital Tokyo Japan; ^2^ Department of infectious diseases Keio University School of Medicine Tokyo Japan; ^3^ Department of Microbiology and Infectious Diseases Toho University School of Medicine Tokyo Japan

**Keywords:** *Bacillus subtilis* subsp., *Bacillus subtilis natto*, bacteremia, natto, pneumonia

## Abstract

A 70‐year‐old immunocompetent male with a history of insomnia presented with pneumonia and bacteremia caused by *Bacillus subtilis*. The patient took benzodiazepines and regularly consumed alcohol and natto (fermented soybeans). Initial antibiotic treatment was not effective, and bronchoalveolar lavage was performed. Bronchoalveolar lavage fluid (BALF) analysis revealed an increased lymphocytes fraction, and *B. subtilis* was detected in the BALF. Whole‐genome sequencing confirmed the congruence of the genetic sequences between the strain in the blood culture of the patient, BALF, and strain isolated from the consumed natto, confirming *B. subtilis* subsp*. natto* as the causative pathogen of pneumonia and bacteremia. Vancomycin followed by levofloxacin and systemic corticosteroid were used to treat the condition. This case highlights community‐acquired pneumonia and bacteremia caused by *B. subtilis* subsp*. natto*, particularly in individuals who consume natto.

## Graphical abstract

We report a rare case of pneumonia and bacteremia caused by *Bacillus subtilis* subsp. natto in an immunocompetent patient. *B. subtilis* subsp. natto is a gram‐positive spore‐forming bacteria and a subspecies of *B. subtilis*. Natto is a Japanese traditional food made by fermenting soybeans with *B. subtilis* subsp. natto which can produce nattokinase.

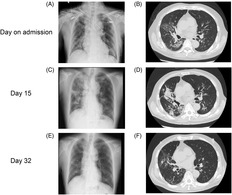

## INTRODUCTION

*Bacillus subtilis* subsp*. natto* is a gram‐positive spore‐forming bacteria and a subspecies of *B. subtilis*. Natto is a Japanese traditional food made by fermenting soybeans with *B. subtilis* subsp. *natto* which can produce nattokinase. Natto is a type of probiotic widely consumed in Japan and generally considered to have beneficial effects on the body. However, there have been several reports of severe infections in patients that consume natto caused by *B. subtilis* subsp. *natto*, particularly in cases of gastrointestinal perforation or immunocompromised individuals.^1^, ^2^, ^3^ On the contrary, there are no reports of community‐acquired pneumonia in an immunocompetent host. We report a case of pneumonia and bacteremia caused by *B. subtilis* subsp*. natto*.

## CASE REPORT

**FIGURE 1:**
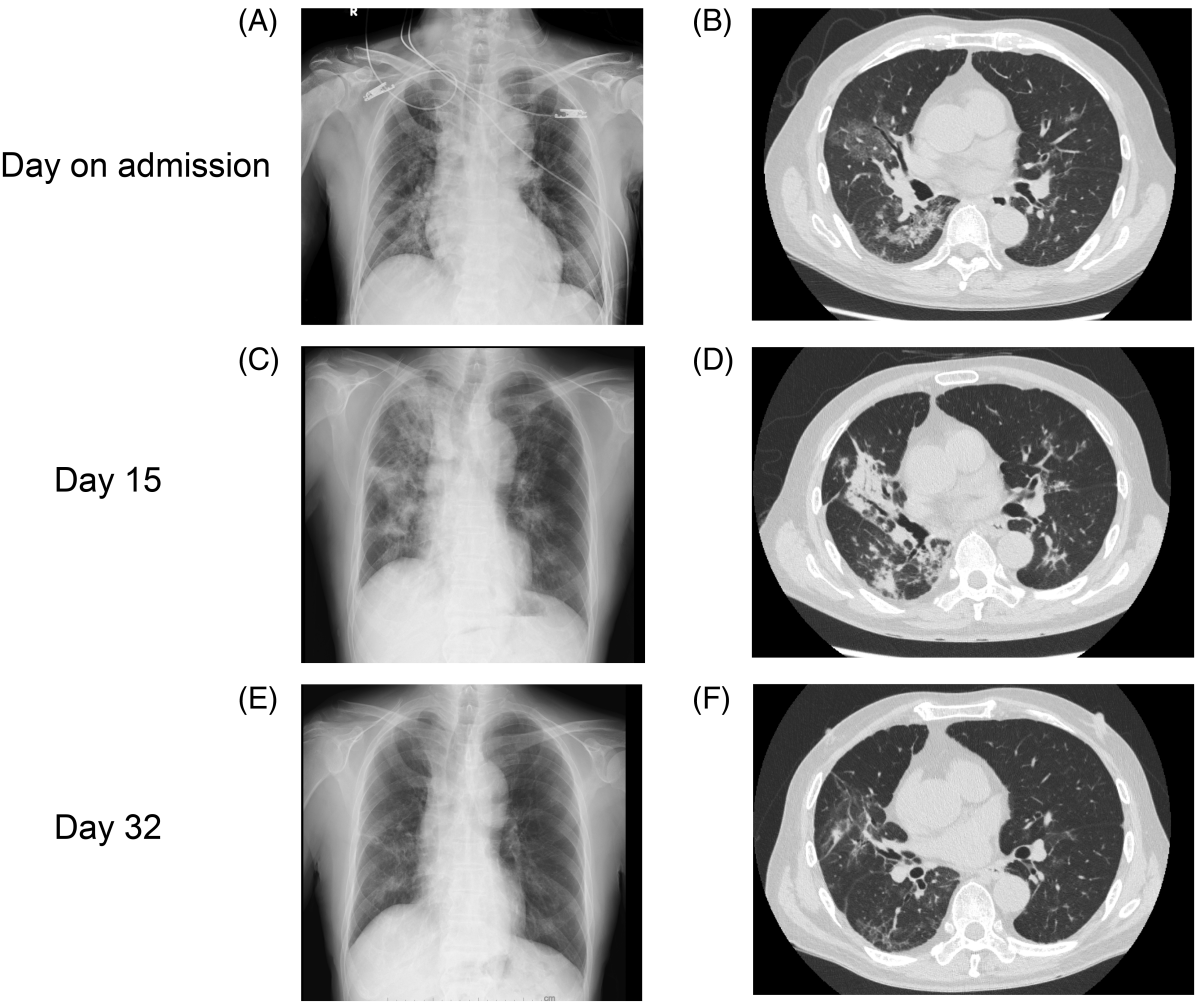
Chest radiograph and computed tomography (CT). Chest radiograph and CT scan showed bilateral ground‐glass attenuation on admission (A, B). Chest radiograph showing shadow exacerbation predominantly in the right lung (C) and CT scan showing consolidation predominantly in the right lung (D) on day 15. Chest radiograph showing decreased shadow (E) and CT scan showing decreased consolidation in bilateral lung (F) on day 32.

A 70‐year‐old male, who regularly took multiple benzodiazepines (triazolam, etizolam, and brotizolam) for insomnia and consumed alcohol daily, presented with a sudden onset of high fever and compromised mobility. Subsequently, the patient was transferred to the emergency room. His vital signs at the time of transfer while receiving 6 L/min oxygen through a reservoir mask were as follows: blood pressure, 119/82 mmHg; heart rate, 99 bpm; body temperature, 39.1°C; respiratory rate, 24 times/min; and SpO_2_, 95%. The blood test findings were as follows: leukocyte, 7900/mm^3^; haemoglobin, 11.8 g/dL; platelet, 157,000/mm^3^; alanine aminotransferase, 17 U/L; aspartate aminotransferase, 59 U/L; C‐reactive protein (CRP), 10.87 mg/dL; prothrombin time‐international normalized ratio, 1.08; and SARS‐COV2 PCR, negative. Chest radiograph and computed tomography (CT) scan (Figure 1A,B) confirmed pneumonia, leading to his admission to our hospital. We started treatments with ampicillin‐sulbactam (12 g/day) and azithromycin (500 mg/day), but the treatment did not improve his condition. *Bacillus subtilis* was detected in the sputum culture and two sets of blood culture samples on the day of admission. Blood cultures were processed through the BD BATEC system (Becton Dickinson, Sparks, Maryland). Gram‐positive rods were detected in one of them and identified as *B. subtilis* via mass spectrometry.

Vancomycin therapy was initiated on day 2. From the blood test, we observe that the treatment resolved the fever and decreased the CRP level but not the oxygen demand of the patient, and shadows were observed on the chest radiograph and CT scan obtained on day 15 (Figure 1C,D). Therefore, we performed bronchoalveolar lavage on day 16. *B. subtilis* was detected in the bronchoalveolar lavage fluid (BALF), and the cell fraction of BALF showed increased levels of lymphocytes (28%) and neutrophils (13%) raising suspicion of *B. subtilis* pneumonia and secondary organizing pneumonia. Based on drug susceptibility results, oral administration of levofloxacin and 1 mg/kg of prednisone per day were initiated, and on day 32, the treatment resolved the condition of the patient as well as the shadows observed on the chest radiograph and CT scan (Figure 1E,F). The patient used to consume 1 pack of natto (fermented soybeans) every day until the day before he was admitted; therefore, we performed draft whole‐genome sequencing of each *B. subtilis* strain isolated from a sample of blood culture (TUM22925), BALF (TUM22926), and the same brand of natto that the patient consumed daily (TUM22927) by using Illumina MiSeq (San Diego, CA). The average sequence depth was 105× for TUM22925, 88x for TUM22926, and 111× for TUM22927. Sequence data are available in the GenBank BioProject under accession number PRJNA1083427. Core‐genome single nucleotide polymorphism‐based analysis demonstrated that the three strains were indistinguishable. All strains possessed genetic elements specific to *B. subtilis* subsp*. natto*, which are not present in *B. subtilis* but only in subsp*. natto*, namely IS*4Bsu1* and IS*256Bsu1*. These elements were analysed by nucleotide BLAST sequencing. There were no single nucleotide polymorphisms within the core‐genome region of these strains compared to those in the reference genome. This region represents 98.3% of the total genome (4,050,056 bp out of 4,105,380 bp). These results confirmed that the genetic elements in natto and patient samples were derived from the same strain of *B. subtilis* subsp*. natto*.

## DISCUSSION

We report a case of pneumonia and bacteremia caused by *B. subtilis* subsp*. natto* followed by suspected secondary organizing pneumonia in an immunocompetent patient.

*B. subtilis* subsp. *natto* is a subspecies of *B. subtilis*, and it is used in the process of making natto. *B. subtilis* is found in the soil and human gut, and it is the major cause of contamination in most clinical cases.^4^ We isolated *B. subtilis* from two sets of blood cultures, and based on this finding, we diagnosed the patient with pneumonia and bacteremia caused by *B. subtilis*. Recent molecular gene analysis has allowed for the identification of *B. subtilis* subsp. natto from other subspecies.^5^ *B. subtilis* subsp*. natto* and other species of *B. subtilis* rarely induce infection; however, there are several reports of infection by *B. subtilis* subsp. natto, such as bloodstream infections and meningitis in natto consumers.^1^, ^2^, ^3^, ^6^ To our knowledge, this case is the first report of community‐acquired pneumonia via this pathogen. The mechanism of pneumonia is unclear. However, there are two possibilities including aspiration of *B. subtilis* subsp*. natto* from the oral cavity or bloodstream infection. The patient took benzodiazepines and alcohol, consequently elevating the risk of developing aspiration pneumonia, and a CT scan revealed no other evidence of infection. Additionally, this was not an immunocompromised or a COVID‐19 patient in whom bacterial translocation occurs occasionally,^1^ and the pathogen was detected in the blood and BALF. For the above reasons, it was suggested that aspiration‐related pneumonia occurred, but as there have been no previous reports on the relationship between aspiration pneumonia and *B. subtilis* subsp. *natto*, we expect further studies on the mechanism of aspiration‐related pneumonia.

The patient developed secondary organizing pneumonia and corticosteroid was used to treat the pneumonia. Organizing pneumonia is a pattern of lung‐tissue repair after injury and secondary organizing pneumonia is attributable to a specific cause such as infection, drug toxicity, inhalation injury, radiation therapy, connective‐tissue disorder, aspiration, or cancer.^7^ Owing to the absence of histopathological confirmation via a transbronchial lung biopsy, a definitive diagnosis of organizing pneumonia was not established. However, imaging findings, BALF, and therapeutic responsiveness strongly suggested organizing pneumonia. To our knowledge, there are no cases of organizing pneumonia after pneumonia owing to *B. subtills* and *B. subtilis* subsp. *natto*. Although detection of this species is generally considered as contamination, the pathogen may be a more common cause of organizing pneumonia in clinical practice.

In conclusion, we present a case of pneumonia caused by *B. subtilis* subsp*. natto* followed by suspected secondary organizing pneumonia in a natto consumer. *B. subtilis* subsp*. natto* could induce pneumonia and bacteremia, and physicians should not immediately assume a case of contamination when *B. subtilis* is detected in natto‐consuming hosts.

## AUTHOR CONTRIBUTIONS

Tetsuo Tani: The patient's physician and primary author. Tomohiro Takehara, Kota Ishioka, and Ayumi Yoshifuji were involved in intensive care management and review of the work and final improvement of the article. Kotaro Aoki performed the analysis of the samples by draft whole‐genome sequencing.

## CONFLICT OF INTEREST STATEMENT

None declared.

## ETHICS STATEMENT

The authors declare that appropriate written informed consent was obtained for the publication of this manuscript and accompanying images.

## Data Availability

Data available on request from the authors.
